# Clinical experiences with intraoperative electrocochleography in cochlear implant recipients and its potential to reduce insertion trauma and improve postoperative hearing preservation

**DOI:** 10.1371/journal.pone.0266077

**Published:** 2022-04-22

**Authors:** Andreas Buechner, Michael Bardt, Sabine Haumann, Gunnar Geissler, Rolf Salcher, Thomas Lenarz

**Affiliations:** 1 Department of Otolaryngology, Hannover Medical School, Hannover, Germany; 2 European Research Center, Advanced Bionics GmbH, Hannover, Germany; University of California Los Angeles, UNITED STATES

## Abstract

Access to low-frequency acoustic information in cochlear implant patients leads to better speech understanding in noise. Electrocochleography (ECochG) can provide real-time feedback about the health of the cochlea during the insertion process with the potential to reduce insertion trauma. We describe our experiences of using this technique. Data from 47 adult subjects with measurable residual hearing and an Advanced Bionics (Valencia, CA) SlimJ (46) or MidScala (1) electrode array were analyzed. ECochGs were recorded intraoperatively via the implant. The surgeon adjusted the course of the electrode insertion based on drops in the ECochG. The final array position was assessed using postoperative imaging and pure tone thresholds were measured before and after surgery. Three different patterns of ECochG response amplitude were observed: Growth, Fluctuating and Total Loss. Subjects in the growth group showed the smallest postoperative hearing loss. However, the group with fluctuating amplitudes showed no meaningful correlation between the ECochG responses and the postoperative hearing loss, indicating that amplitude alone is insufficient for detecting damage. Considering the phase of the signal additionally to the amplitude and reclassifying the data by both the phase and amplitude of the response into three groups Type I–Type III produced statistically significant correlations between postoperative hearing loss and the grouping based on amplitude and phase respectively. We showed significantly better hearing preservation for Type I (no drop in amplitude) and Type II (drop with a concurrent phase shift), while Type III (drop without concurrent phase shift) had more surgery induced hearing loss. ECochG potentials measured through the implant could provide valuable feedback during the electrode insertion. Both the amplitude and phase of the ECochG response are important to consider. More data needs to be evaluated to better understand the impact of the different signal components to design an automated system to alert the surgeon ahead of damaging the cochlea.

## Introduction

The preservation of any low frequency residual hearing in patients who undergo cochlear implant surgery has become an important consideration in electrode array design and surgical technique. Low-frequency hearing is associated with improved hearing performance, especially in difficult listening environments (i.e., the cocktail party effect). Access to low-frequency acoustic information in cochlear implant patients can enhance the segregation of competing voices, which leads to better speech understanding in noise, and the benefit of Electric Acoustic Stimulation (EAS) has been shown in numerous studies, e.g. [1–4]. However, damage to the cochlea during surgery does occur and reported rates of preservation of residual hearing vary with between 50 to 90% of subjects retaining usable acoustic hearing [4–8]. Due to this large variability in hearing preservation outcomes, many potential cochlear implant candidates show reluctance to undergo surgery. Therefore, possibilities to reduce the potential trauma need to be explored. Tools which can provide real-time feedback about the health of the cochlea during the insertion process may enable the surgeon to adapt the insertion path to avoid any trauma and provide a vital learning tool for any mistakes. Electrocochleography (ECochG) is one approach to providing this feedback.

Electrocochleography is the process of recording electrical potentials generated in the cochlea and from the auditory nerve in response to an acoustic stimulus. It consists of four waveforms which can be separated using filtering and the appropriate time window. The cochlear microphonic (CM) is an alternating current voltage that reflects the instantaneous displacement of the basilar membrane and comes from active movements from the outer hair cells [9]. Summating potential (SP) is a complex response comprised of several components which is recorded as a rectified, direct current similar to the stimulus envelope. It was thought to come primarily from the inner hair cells [10] while newer research suggests contribution also from the outer hair cells and the auditory nerve [11]. The action potential (AP) reflects the cochlear nerve activity and consists of the compound action potential (CAP) and auditory nerve neurophonic (ANN). The CAP is observed in response to the onset/offset of the acoustic stimulus and the ANN reflects the phase-locked response of auditory nerve fibres to the stimulus at low frequencies [12].

The first cochlear potentials were recorded from cats by Wever and Bray in 1930 but not successfully recorded in humans until 1960 [13, 14]. Today, ECochG is used clinically for threshold estimation, diagnosis of auditory neuropathy and Meniere’s and for the intraoperative monitoring of cochlear function during surgical procedures such as cochlear implantation or stapedectomy [15]. Adunka et al. (2006) recorded the first usage of ECochG during cochlear implant surgery (N = 1) [16]. Responses were measured at different surgery stages with a Tiptrode electrode placed in the external ear canal. They found that the mastoidectomy, drilling, scalar opening and large cochleostomy did not diminish the CM amplitude, but the electrode array insertion did. Further work showed that the intraoperative ECochG recorded using an extracochlear electrode could be an indicator of cochlear health during implant surgery and that responses were related in some way to postoperative thresholds [17–20]. In 2015 Campbell et al. showed the feasibility of measuring the CM using the cochlear implant’s amplifier and back telemetry, greatly reducing the complexity of making such a recording [21]. Responses recorded using an intracochlear electrode correlate well with extra-cochlear recordings and are even larger in amplitude [22, 23].

Further research was undertaken to establish if the observed variations in ECochG responses could be related to postoperative residual hearing or even speech perception outcomes [24–26]. Campbell et al. (2016) reported that subjects with a preserved CM at the end of the surgical procedure had better low frequency hearing after surgery [21]. They hypothesized that contact with the basilar membrane towards the end of the insertion was the most likely cause of any hearing loss. Dalbert et al. (2016) also reported that out of six subjects who had a loss of residual hearing > 11dB, four showed a reduction in either high or low frequency ECochG responses [27]. In a subsequent publication, Dalbert et al. (2018) combined data from their previous publications to show a correlation between the low frequency extracochlear ECochG response and the postoperative pure tone audiogram (r = -0.44, p = 0.055) [28]. They also showed a significant difference in the degree of hearing loss between groups with and without a decrease in ECochG response after array insertion. Guiardina et al. (2019) however, found that changes in magnitude of the ECochG response alone did not account for hearing preservation rates, but phase, latency, and neural contribution should be considered [23]. This use of phase as well as amplitude to categorise responses was also explored by Koka et al. (2018) in a study of the use of ECochG responses to estimate the scalar position of the array [29].

Conversely, a number of studies have shown no relationship between intraoperative ECochG responses and thresholds [22, 30, 31]. Although O´Connell did not observe a significant correlation between ECochG thresholds obtained immediately after insertion and postoperative audiometric thresholds at activation, they did note that the difference between intraoperative ECochG thresholds and postoperative audiometric thresholds was significantly lower (i.e., better) for electrodes completely located in scala tympani. They suggest that their data supports the notion that changes in cochlear physiology occur in the time period between electrode insertion and activation and are more pronounced for electrodes that translocate into the scala vestibuli. In a small sample of 10 subjects Haumann et al. (2019) found no correlation between intraoperative ECochG recordings to postoperative pure tone thresholds [22]. However, intraoperative ECochG thresholds correlated highly with preoperative audiometric thresholds. ECochG recordings made via the implant postoperatively correlate strongly with pure tone thresholds measured at the same time [22, 30, 32, 33]. This indicates that timing of the threshold measurements in relation to the ECochG measurements is important as we know that inflammatory processes occurring post-surgery can further reduce hearing thresholds.

We started using ECochG in 2016 to systematically collect inner ear potentials during the electrode insertion process in as many subjects as possible who had measurable residual acoustic hearing. These potentials were then related to the pre- and postoperative hearing thresholds and the final position of the electrode array of these subjects. The main objective of this study was to describe our experiences of using this technique in a large sample of Advanced Bionics (Valencia, CA) CI recipients with some measurable hearing in the implanted ear.

## Methods

ECochG was used as a tool to guide the surgeon during the insertion of the CI array. Sharp drops in the ECochG responses were communicated to the surgeon, who adjusted the course of the electrode array, aiming to restore the amplitude of the continuously measured intracochlear potentials. The final position of the electrode array was assessed using postoperative image analysis and pure tone thresholds were recorded before and after surgery. Further analysis was performed to look for any features of the ECochG response that could possibly indicate a greater loss of residual hearing post-surgery. The hypothesis was that by using this technique the insertion process could be managed with less trauma and subsequently result in better preservation of residual acoustic hearing.

### Subjects

64 subjects aged 18 and above were included in the study. These were patients who had opted for an Advanced Bionics (Valencia, CA) cochlear implant and had measurable residual hearing (HL < = 80dB) for at least one frequency. All subjects who fulfilled those inclusion criteria were asked to participate in the study one or two days before the surgery.

Subjects followed routine surgery and follow up procedures. Additional to the routine measurements, ECochGs were recorded intraoperatively via the implant. For the following analyses, 17 of the 64 subjects were excluded as their intracochlear potentials were measured with the much slower 1st generation ECochG system which is not capable of providing real-time feedback. So, data from 47 (numbers 18–64) subjects are presented in this paper.

One subject had a MidScala electrode array and all others received a SlimJ electrode array. Age at implant ranged from 19.5 years to 84.8 years with a mean of 56.9 years. Two hearing losses were acute, six unknown and four had a hearing loss since childhood. The remaining 35 subjects had a progressive hearing loss. Most hearing losses were of unknown cause (25), six losses were hereditary, and seven patients experienced sudden hearing loss. Other etiologies for individuals were Cogans syndrome, Ushers syndrome, noise exposure, sepsis, measles, mumps, trauma, legionella disease and oxygen deficiency. Duration of hearing loss prior to implantation ranged from 1.7 to 50.3 years with a mean duration of 22.1 years.

The study was approved by the ethics board of the Medical University Hannover with Nr 2867–2015 and was conducted in accordance with the principals laid out in the Declaration of Helsinki. All subjects signed an informed consent for participation and to enable their data to be included in this study.

### Audiological assessment

Pure tone thresholds for 125, 250, 500, 1000, 1500, 2000, 4000 Hz were measured under headphones using calibrated clinical equipment. This was done prior and 4–6 weeks after surgery at the first fitting of the speech processor.

### Intracochlear ECochG recording setup and surgical procedure

Intracochlear ECochG recordings were conducted through an Advanced Bionics HiRes90K or Ultra CI system (Advanced Bionics LLC, Valencia, CA). Advanced Bionics research ECochG software running on a laptop was used for stimulus generation and data collection. The laptop was connected to the CI through the Clinical Programming Interface 3 (CPI3) and the NaidaCI Q70 speech processor. The amplifier in the implant was configured to have a gain of 1000. The sampling rate was 9,280 Hz. The low pass filter was set at 5,000 Hz. The most apical contact of the HiFocus Mid-Scala and SlimJ electrode arrays was used as the recording electrode, the case as reference electrode.

The acoustic stimulus was generated by a Behringer soundcard and presented through a sterilized ER-3A insert earphones (Etymotic Research Inc., Elk Grove Village, IL, USA). As acoustic stimulus, a 53 ms long sinusoidal tone burst at 500 Hz with a level of approximately 110 dB SPL was used. The CPI3 received an external trigger by the soundcard to synchronize stimulus generation and ECochG recording through the CI. Consecutive buffers were presented with alternating polarity and the cochlear microphonic (CM) were computed by subtraction of the two recordings. The recordings were acquired continuously during insertion of the CI electrode and averaged until a signal to noise ratio of 12 dB was reached or a maximum number of 40 averages reached (with significant responses resulting in up to 8 measurements per second). The CM is transformed to the frequency domain with a FFT (Fast Fourier Transform). The response magnitude is derived from the FFT bin containing the stimulus frequency. The noise floor is estimated by taking the mean of a few surrounding bins.

The cochlea was approached using a standard posterior tympanotomy and the round window was opened for manual insertion of the electrode array by making a slit through the round window membrane. The electrode array was inserted slowly over 1–2 minutes as it is our standard clinical practice in hearing preservation surgeries. ECochG responses were measured continuously during the insertion and the surgeon was informed verbally about any changes. There was no strict procedure how to react to changing ECochG amplitudes, but generally the surgeons behaved as follows:

- When the amplitude dropped during advancing the electrode array, the insertion was paused, or the array was pulled back slightly.
- When the response recovered completely or partially, the surgeon continued to insert the array, maybe with a different angle or more carefully than before.
- When the amplitude dropped again during insertion and only 1–3 contacts were outside the cochlea, the electrode array was pulled back again for amplitude recovery and left in place at that position.
- If the amplitude drop occurred with 13 or less contacts in the cochlea and restoring the ECochG amplitude was not possible, full insertion was performed anyway.

### Image analysis

Advanced image processing was conducted for estimating the angular insertion depth and scalar location of the electrode. The angular insertion depth was calculated in a coronal plain projection with a 0° reference line projected through the middle of round window and the centre of the modiolus [34]. Another line was projected from the center of the modiolus and the center of the most apical electrode contact. The angle of rotation between the two lines was the estimated angular insertion depth.

For the assessment of the scalar location of the electrode array, two different methods conducted by two independent experts were applied: The first observer applied the image fusion (co-registration) technique of preoperative MRI and postoperative CT scans as described by Dees et al. (2016) and Dietz et al. (2016) [35, 36]. The second observer applied a refined active shape model for adapting a high-resolution cochlear model to a conventional CT scan. The active shape model is derived from 35 microCT scans of different human temporal bones and is embedded into a research software that can predict the size and morphology of the patient’s cochlea. The individual electrode array contacts are automatically located and a 3D model representing the true form and size of the actual electrode array (SlimJ or Mid Scala) is presented within the 3D model of the patient’s cochlea. Drops in CM amplitude could also be explained by exceeding the characteristic frequency (CF) of the stimulating electrode and thereby increasing the distance between the stimulus generator and the measuring electrode. Based on the calculated insertion angle and the work by Stakhovskaya et al., (2007) the CF of the most apical electrode contact was estimated [37].

## Results

**Fig 1:**
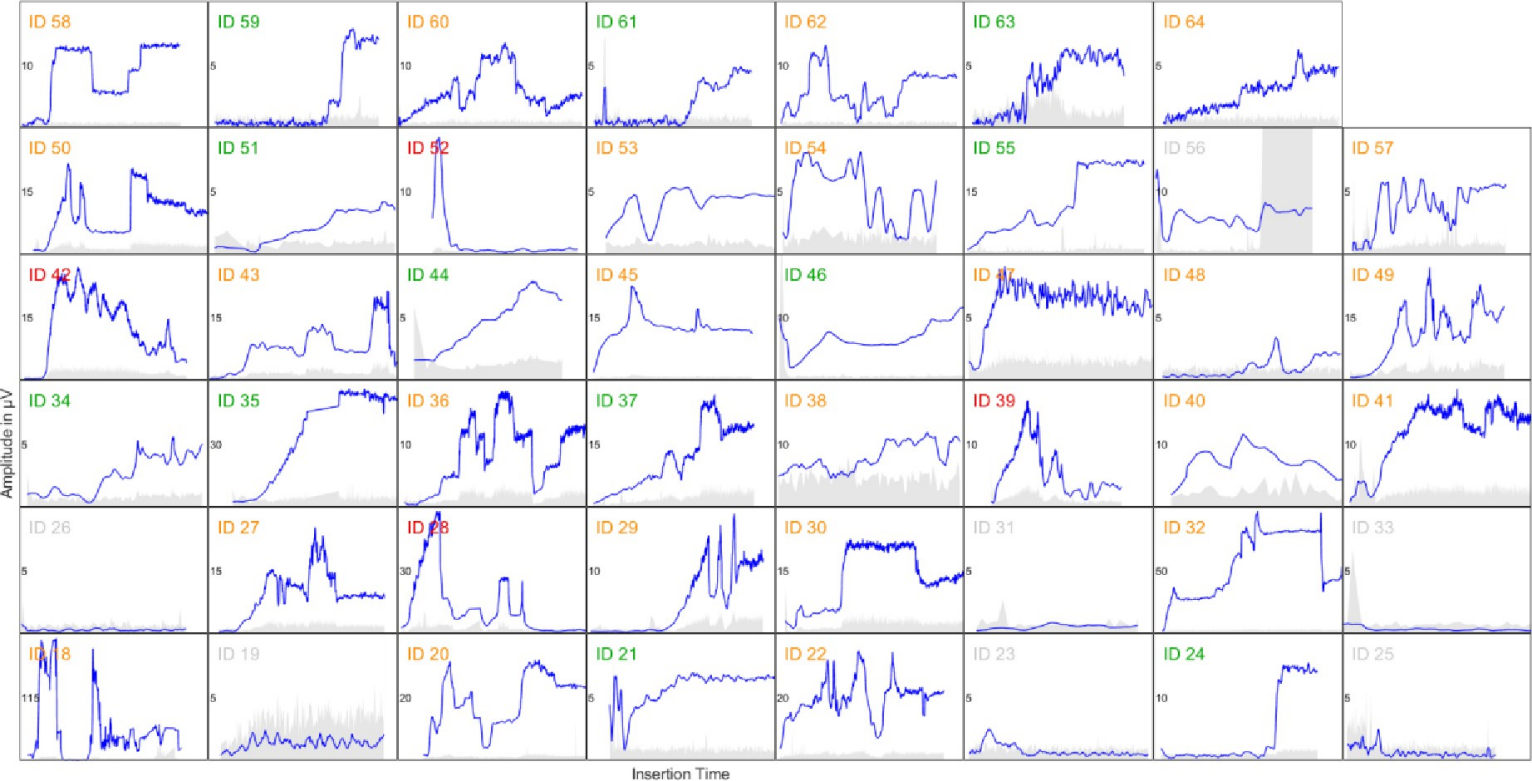
Cochlear microphonic recordings for each subject made intraoperatively via the cochlear implant showing the large variability in responses. Total amplitude of the responses ranged from ~5 μV to over 200 μV (blue line). Light grey area shows the noise floor. The subject ID in each axis is coloured according to the group categorisation (green = Growth, yellow = Fluctuating, red = Total Loss, see also Fig 5).

In 40 out of 47 subjects Cochlear Microphonics above the noise floor could be measured successfully. The intra-OP monitoring results show a large variability over all subjects (Fig 1). For example, subject ID 35 shows an ideal response with a constantly rising amplitude. Subject ID 28 shows rising amplitude in the beginning but then a sudden drop. The surgeon tried unsuccessfully to recover the responses but at the end, amplitude was not above noise floor. In subject ID 49 amplitude was rising in the beginning but after a sudden drop, adjustments by the surgeon were made, resulting in some up and downs. The amplitude at the end was still measurable and might have been affected (in a positive or negative way) by the surgeons handling in response to the monitoring.

### Imaging results

**Fig 2:**
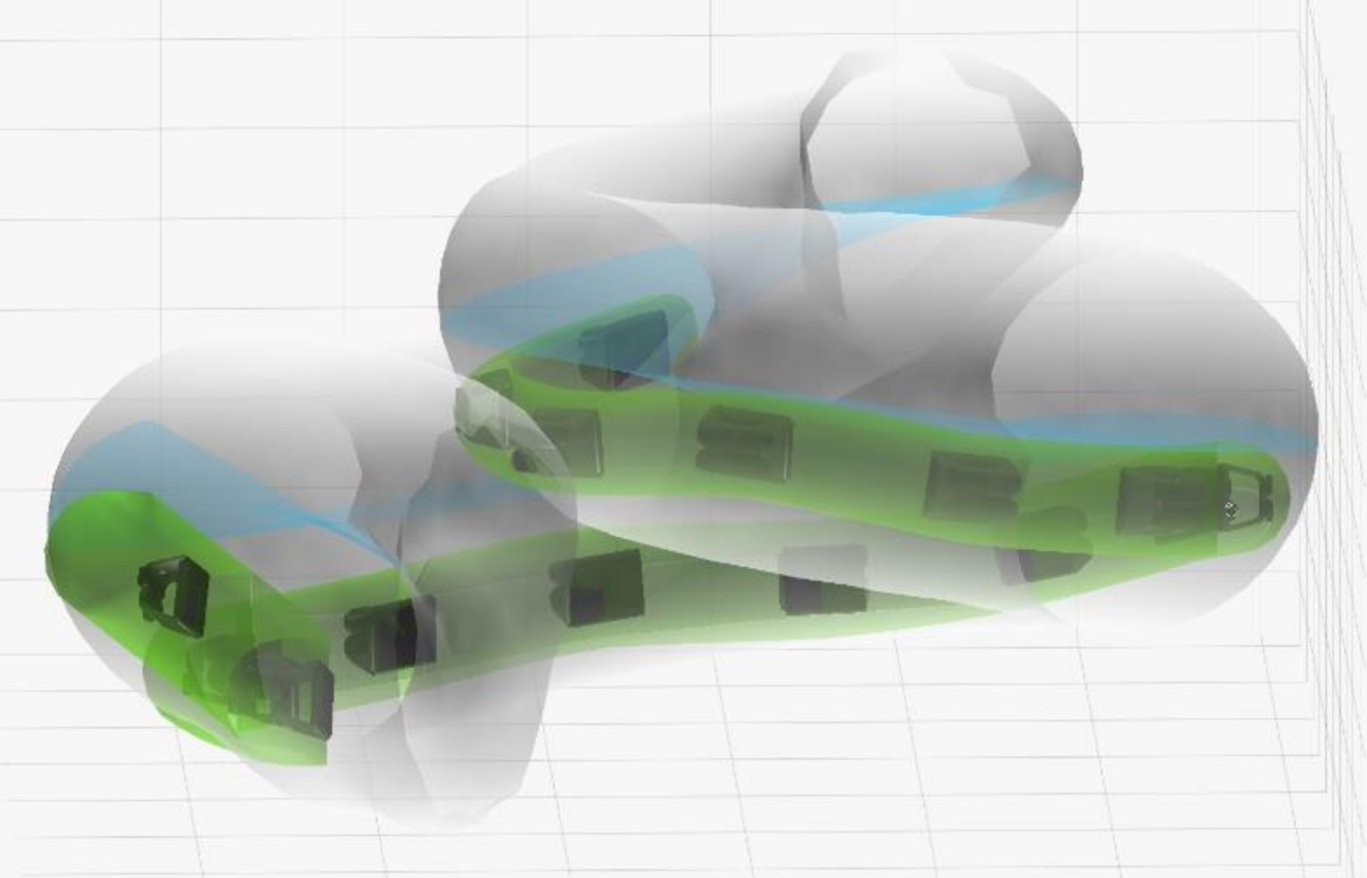
3D visualization of cochlea and electrode position from subject ID 28, showing a potential soft contact with the basilar membrane and some slight buckling in the middle part of the array.

**Fig 3:**
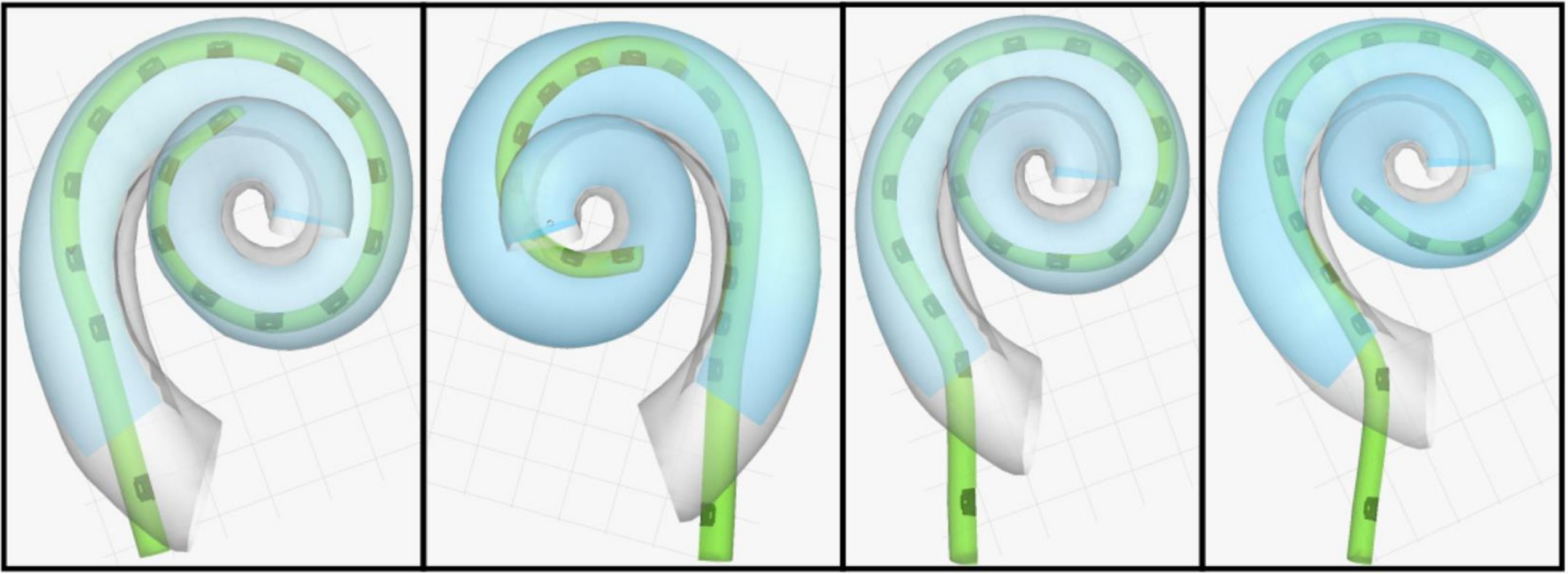
Top view from left to right. ID 24 (Scala tympani without touching the basilar membrane, relatively deep insertion in a small cochlea); ID 27 (translocation of MidScala array); ID 28 (soft contact with the basilar membrane and some slight buckling in the middle part of the array); ID 35 (good position, little less than full insertion).

In 46 cases (ID 63 was not available), post-operative CT scans and preoperative MRI images were available. The insertion angle, the distance of the blue marker to the round window and potential translocations were analyzed. In Fig 2 the 3D visualization from subject ID 28 is shown as an example. In Fig 3 the top view of four different insertions is shown.

The mean insertion angle was 375° (standard deviation: 45°, range: 290 to 500°) which is a little less than the 400–420° which is usually reported for the SlimJ. In 38 subjects the blue marker was 1 mm or more outside the round window, and in 13 subjects it was 3 mm or more.

There was only one clear translocation with the MidScala electrode (ID 27) at around 100° from scala tympani to scala vestibuli. The ECochG response dropped during the last part of the insertion from 25μV to 8μV but was still measurable.

There were four additional subjects where the image analysis indicated potential contact to the basilar membrane (ID 28; ID 31; ID 54; ID 62). In subject ID 31 no responses at all could be measured but in the other two, the amplitude dropped to noise floor during the insertion but recovered and was measurable at the end (Fig 1).

In some cases, the most apical electrode contact, which is used to measure the potentials, reaches or even passes the characteristic frequency (CF) of the stimulation frequency, usually 500 Hz. In those cases, a drop in amplitude could be caused by the increasing distance to the signal generator. Sometimes the electrode array was inserted and pulled back a little because of a drop in CM amplitude. So regarding the postoperative CBCT analyses, we can only report on the final insertion depth—not the maximum insertion depth theoretically reached during surgery.

In two implantations (ID 24 and ID 28) the final position corresponded to a CF smaller than 500 Hz (448 Hz and 489 Hz respectively). No loss in CM amplitude was observed for ID 24. In ID 28 a drop in amplitude was observed and this was accompanied by a large post-operative hearing loss.

In four implantations (ID 18, 33, 59 and 62) the final position corresponded to a CF between 500 Hz and 600 Hz (530 Hz, 530 Hz, 596 Hz and 596 Hz). As these values are only estimations based on Stakhovskaya [37], this would still give the possibility that a potential drop in amplitude could be explained by actually reaching or surpassing the CF. In ID 18 the CM dropped significantly accompanied by phase changes. As this subject had good hearing preservation (20 dB LF HL after 4 weeks, -1.7 dB after 4 months) this could indicate that the CM loss was caused by passing the CF or the area of signal generation. In ID 33, only noise was measured, in ID 59 the CM had a growing profile and in ID 62 fluctuating responses were observed, and the average LF HL after four weeks was 16.7 dB.

### Hearing preservation

**Fig 4:**
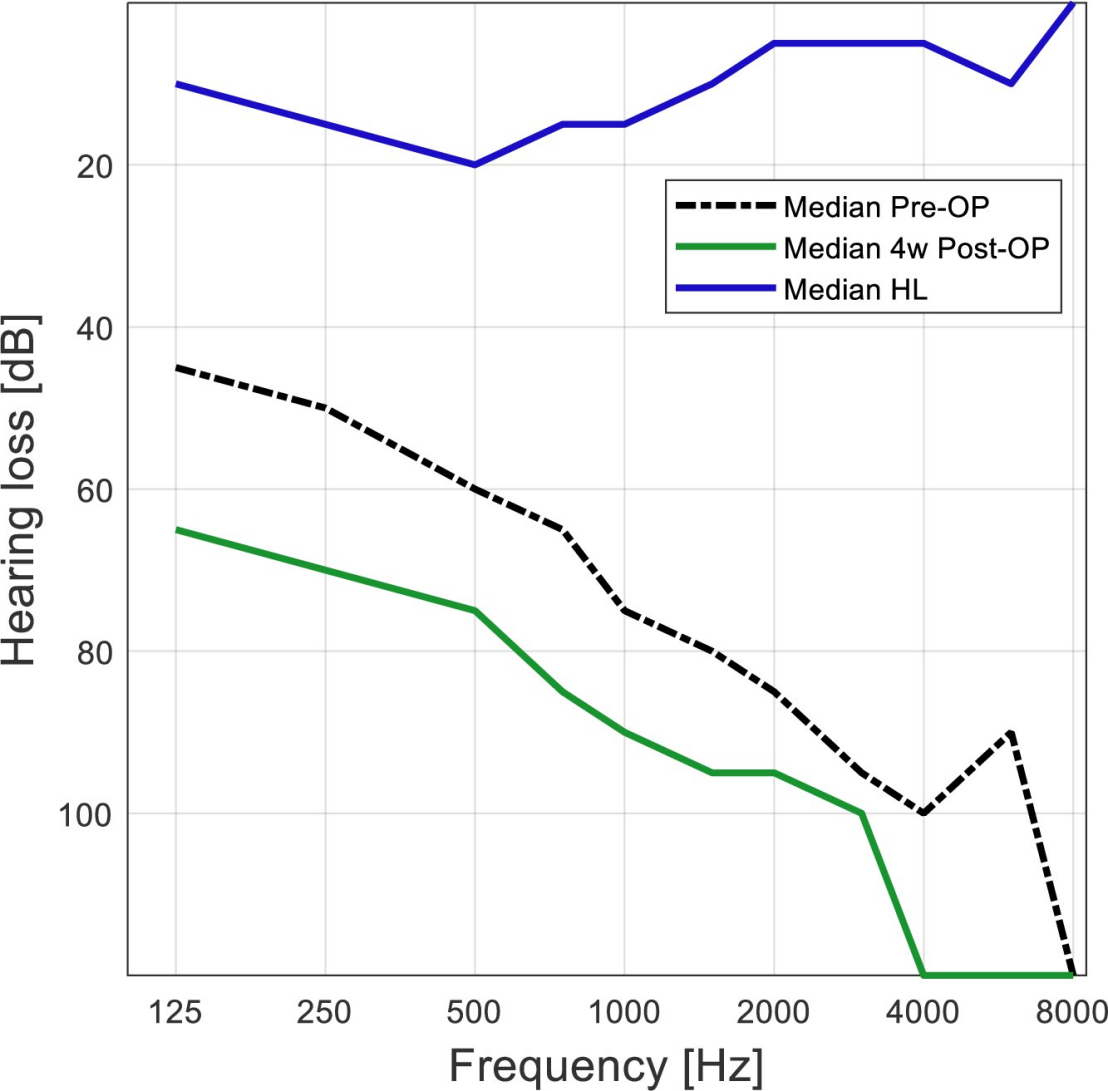
Median hearing loss, calculated as the difference between pre-operative hearing and 4 weeks post-surgery, at each frequency for 47 subjects.

Residual hearing was measured 4 weeks and 4 months after surgery. As the audiograms from the 4 months appointment were missing for 6 subjects, we decided to only evaluate the 4-week data. In Fig 4 median thresholds for all subjects are shown and the hearing loss derived from them.

However, we mainly observed three different patterns: Growth (with no large drops during the insertion), Fluctuating (where some larger drops were observed but by the end of the insertion the response was still present) and Total Loss (where a response could be measured during insertion but not at the end).

**Fig 5:**
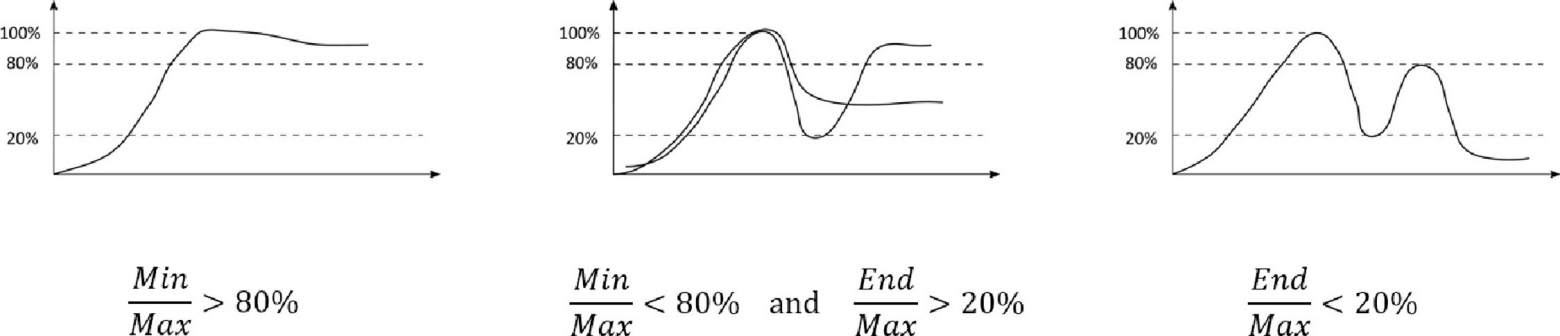
Cochlea microphonic waveform examples for each of the Growth (n = 11), Fluctuating (n = 25) and Total loss (n = 4) groups and the threshold calculations used to define them.

To categorize the insertion patterns objectively, we set up some arbitrary threshold values based on the maximum amplitude measured during insertion (max), the minimal amplitude following max (min) and the end amplitude when insertion and coiling is finished (end) (Fig 5). Weder et al. [38] further investigated ECochG events which predicted best the post-operative hearing loss.

**Fig 6:**
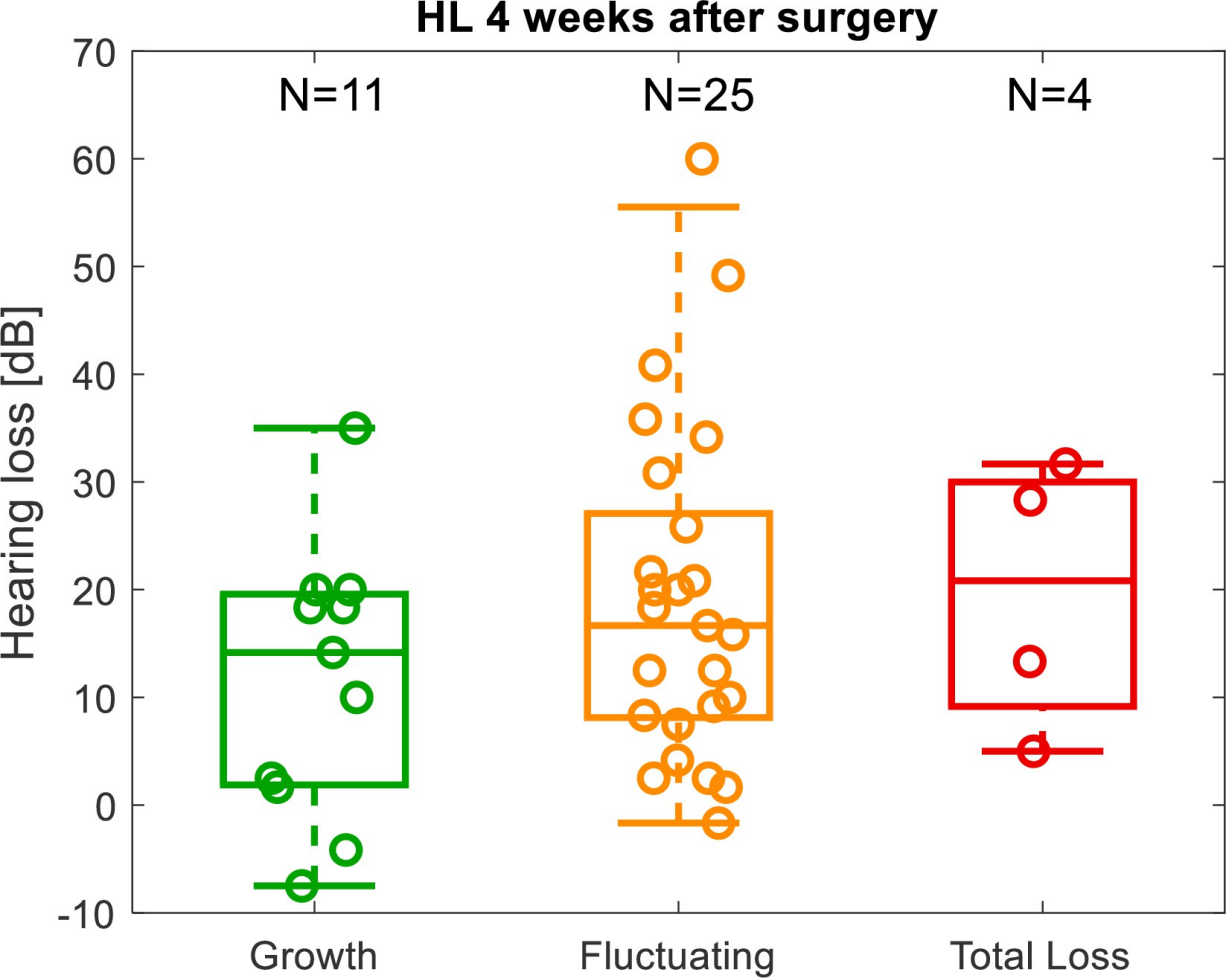
Average low frequency hearing loss after 4 weeks for the three groups of subjects categorized by the intraoperative cochlear microphonic amplitude.

The LF PTA HL for the subjects in each group are shown in Fig 6. They were compared using an Kruskal-Wallis rank sum test but there were no significant differences (p = 0.42). However, in the Growth group only one of these subjects had a loss of more than 20 dB.

Also we computed the correlations between several variables, but no significant relationships were observed (see Table 1).

**Table 1:** Correlation analysis between several intra-operative measures and audiometric/outcome measures. A_max_ is the maximal CM amplitude during insertion. A_min_ is the minimal CM amplitude after A_max_. A_end_ is the final CM amplitude at the end of insertion. Low frequency HL is the average (125 Hz– 1.5 kHz) increase in thresholds 4 weeks after surgery compared to pre-operative.

Variable 1	Variable 2	Pearson correlation coefficient	p
Pre-OP threshold at 500Hz	A_max_	0.12	0.47
A_max_	Monosyllables score with CI	0.23	0.17
A_min_/A_max_	Low frequency HL	-0.28	0.081
A_end_/A_max_	Low frequency HL	-0.12	0.46

Koka et al. (2018) and Giardina et al. (2019) both showed that the phase information of the response might contain useful information [23, 29]. A CM drop might not come from actual damage/trauma but only from a movement of the recording electrode. Therefore, the data was reclassified using the approach adopted by Giardina et al. (2019), where a drop which concurs with a larger phase shift is potentially not connected to trauma.

◦ Type I: no drop > 5 dB
◦ Type II: drop > 5 dB with concurrent phase shift
◦ Type III: drop > 5 dB without concurrent phase shift

**Fig 7:**
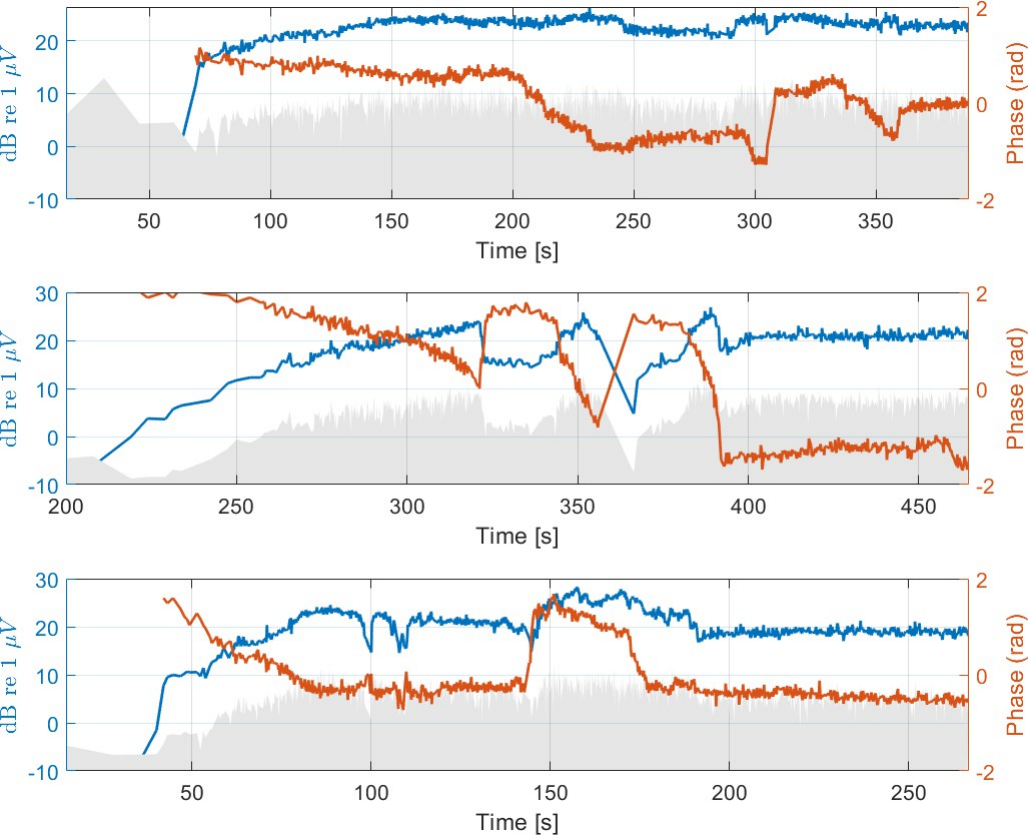
Cochlear Microphonic amplitude (blue) and phase (red) over insertion time. The top panel shows a Type I example where the amplitude does not drop by more than 5 dB. In the middle panel (Type II) each drop is accompanied by large phase changes. The insertion in the lower panel is classified as Type III because the two drops around an insertion time of 100 s don’t come with significant phase changes.

Fig 7 shows one example measurement for each insertion type.

**Fig 8:**
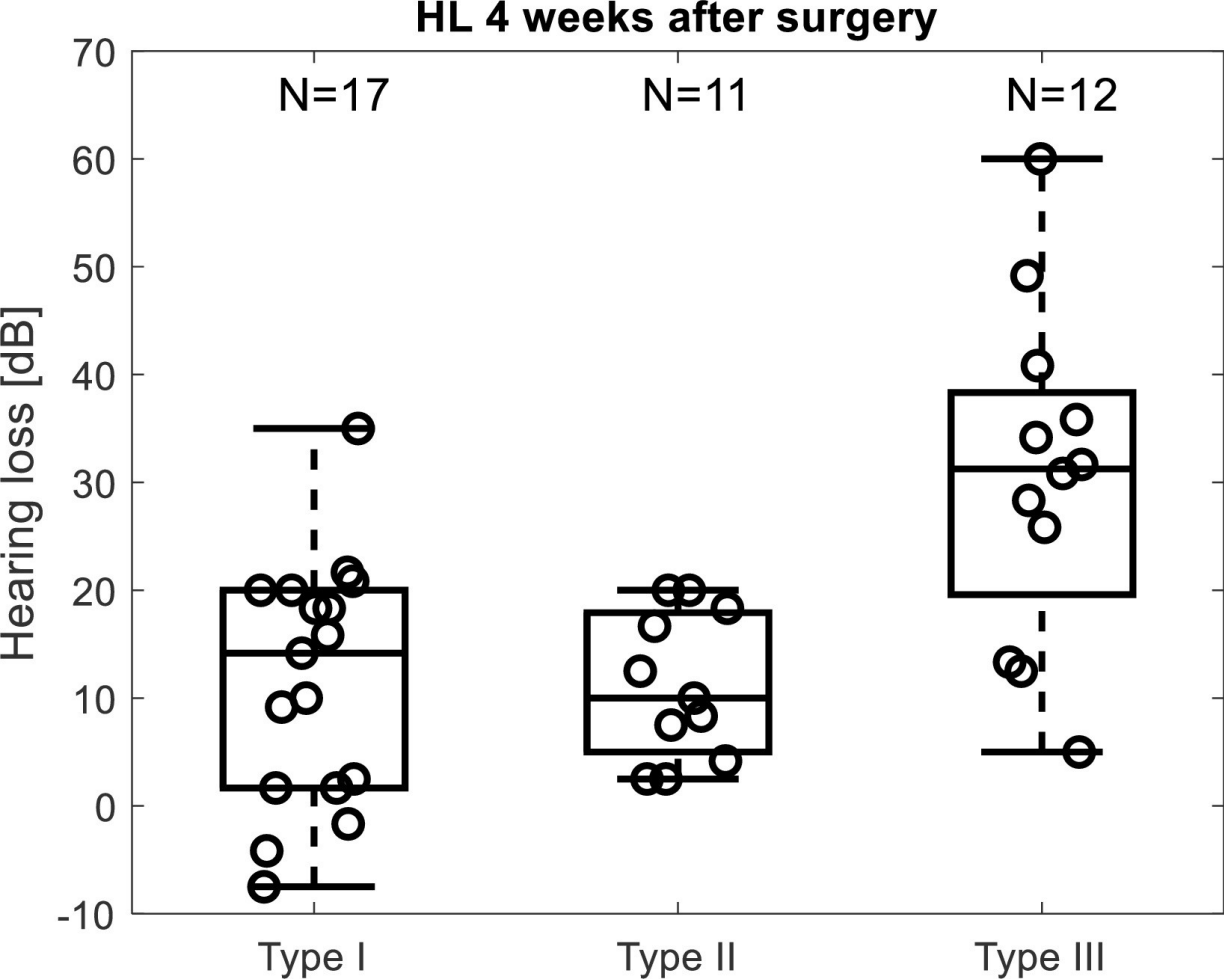
Average low frequency hearing loss after 4 weeks for the three groups of subjects with no drop > 5 dB (Type I), a drop > 5 dB with concurrent phase shift (Type II) and a drop > 5 dB without concurrent phase shift (Type III).

Fig 8 shows significantly worse hearing preservation for Type III compared to both other Types (Kruskal-Wallis rank sum with multiple comparison test, p = 0.005 and p = 0.008) while there was no difference between Type I and Type II (p = 0.983).

To ensure that the worse hearing preservation in the Type III group was not an effect of preoperative residual hearing, the pre-operative hearing thresholds for the three groups were compared. There were no significant differences (unpaired t-test, Type I vs Type III: p = 0.87, Type II vs Type III: p = 0.28).

## Discussion

Our findings show the feasibility of measuring ECochG potentials through a cochlear implant device from inside the cochlea. Responses were recorded from 85% of the subjects. Missing responses in the other seven subjects could be attributed to poor residual hearing, bad acoustic coupling of the speaker in the outer ear canal, fluid in the middle ear, kinking of the sound tube or high impedance of the ground electrode used for recording the responses (the case electrode). Making measurements through the implant electronics instead of using external measurement equipment required little additional hardware and the setup in the operating theater was comparatively simple. This allows for its widespread use as a routine clinical tool. ECochG responses recorded from inside the cochlea are significantly larger than externally measured potentials and this compensates for the lower resolution of the integrated amplifier of the implant electronics compared to an external electrodiagnostic system [22]. The ease of use due to the simpler setup and the more favourable recording site compared to an extracochlear ECochG setup, outweigh the disadvantage of having inferior amplification hardware. It is also worth mentioning that the integrated measurement capability opens up the possibility of making postoperative ECochG measurements at regular patient aftercare visits, allowing for an easy longitudinal follow-up of the inner ear potentials.

It is important to consider that the study was not purely observational. By reacting to the amplitude changes during insertion to attempt to avoid cochlear trauma, a simple correlation of the intraoperatively measured amplitudes and the hearing preservation cannot easily be made. Research has also shown, that even in purely observational trials ECochG amplitude could fluctuate [28, 38]. But in general when looking at the amplitude progression during the insertion process, we saw 3 different courses: (1) the ECochG amplitude increased until the end of the insertion, (2) the amplitude showed intermediate drops but recovered as the surgeon changed the insertion trajectory, or (3) the amplitude fell, and it was not possible to recover the potentials.

From looking at the ECochG amplitude alone, we can conclude that the subjects with a more or less constant increase in the potentials during the insertion process (group “growth”) showed the smallest loss of their acoustic hearing pre- vs. postoperatively. This is also in line with previous work by Campbell and colleagues [21]. However, considering the group with fluctuating responses during electrode progression, no clear statement can be made. In fact, the group with fluctuating amplitudes showed no meaningful correlation between the ECochG responses during insertion and the hearing loss at all. This is partially also due to the limited knowledge about the nature of the fluctuations. In most cases it could not be reconstructed, which fluctuations might be caused by a movement of the recording electrode and which are connected to actual trauma. So this research question can not yet be answered. The ECochG response was also of limited use when considering the array position within the cochlea, based on the imaging results.

Amplitude changes during the insertion could result from the change of the recording site in relation to the generator as well as from cochlear trauma. It is therefore necessary to consider more parameters than just the amplitude of the CM. Koka et al. (2018) first described a method to take the relation of the phase between the acoustic stimulus and the recorded signal into account [29]. The idea behind considering the phase is that while inserting the electrode array into the cochlea, the measurement probe, i.e., the most apical electrode contact, approaches the signal generator leading to an increasing ECochG signal up to the point where the tip of the electrode has reached the optimal placement to record the potentials, i.e., being closest to the generator. Further advancement of the electrode increases the distance to the signal generator again, but as the signal is now being picked up from “behind” the generator, thus the phase of the measured response significantly changes compared to the one of the acoustic stimuli. This hypothesis is supported by work of Kohllöffel et al. (1970) who recorded ECochG potentials in guinea pigs at different locations inside the cochlea [39]. In his work, he demonstrated phase shifts of up to 180 degrees and decreasing CM amplitudes when the recording electrode passed the region with the according characteristic frequency (CF) of the acoustic stimulus. There were only two subjects where–based on our postoperative image analyses—the CF was passed and in one the CM was unchanged and in the other, where the CM was lost, a large hearing loss was observed.

It needs to be considered though that in our subjects the intracochlear region with a CF equal to the stimulus frequency was hardly reached, although Bester et al. [40] have shown that in individuals with substantial hearing loss the CF can be shifted significantly basal-ward. The adaptation in surgical procedure resulting from the ECochG feedback meant that the blue marker at the electrode array, which indicates full insertion, was outside the cochlea for most of the subjects and the insertion angle reduced. Often the CM signal dropped during the last few mm of insertion and the electrode was pulled back a little. If the CM signal recovered, the array was kept in that position in hope of better hearing preservation at the expense of full insertion up to the marker. However, it could be possible that more basally located isolated regions of residual outer hair cells are responsible for the patterns we see. It could be hypothesized that these patches of outer hair cells are still generating a measurable potential even though they are not in the optimal CF region corresponding to the stimulus signal.

The partial insertion in cases of a recovering CM response, where only 1–3 electrode contacts have been left outside the cochlea, is motivated by the increasing chance of cochlear trauma with insertion depth [41]. In cases with good low-frequency hearing, it might be a good decision to withdraw from inserting the last few contacts into the cochlea, thereby increasing the chance of preserving the acoustic residual hearing. Future analysis of larger data sets have to show, if our individual approach of partial insertion really is beneficial for the subjects. In case of post-operative hearing loss, the electrode array can be fully advanced into the cochlea during a small second surgery.

Taking both the phase and the amplitude into account allows for a discrimination between two scenarios: (1) the ECochG amplitude drops because the basilar membrane is being touched or even damaged or (2) the ECochG amplitude drops because the generator has just been passed and damage is unlikely. Grouping our data according to this extended notion gave us far better stratification and resulted in a statistically significant difference in post-operative surgery-induced hearing loss between the groups. We do not claim to have the perfect analysis method or can explain everything which happens during and (maybe more importantly) after the surgery to the inner ear. However, considering not only the amplitude of the CM response but also the phase information, seems to reveal important information which needs to be considered in the interpretation of the results. Future research should continue to focus on this approach to optimize the usefulness of ECochG in preventing intraoperative cochlear damage during electrode insertion.

## Conclusion

ECochG potentials measured through the CI electrode array can give valuable feedback to the surgeon to determine when trauma is possibly impending. Both the amplitude and phase of the EochG response are important to consider. More data needs to be evaluated to better understand the impact of the different signal components to design an automated system to alert the surgeon ahead of damaging the cochlea.

## Supporting information

S1 Data (XLSX)

## References

[1] Von Ilberg C, Kiefer J, Tillein J, Pfenningdorff T, Hartmann R, Stürzebecher E, et al. Electric-acoustic stimulation of the auditory system. New technology for severe hearing loss. ORL. 1999;61: 334–340. doi: 10.1159/000027695 10545807

[2] Gantz BJ, Turner C, Gfeller KE. Acoustic plus Electric Speech Processing: Preliminary Results of a Multicenter Clinical Trial of the Iowa/Nucleus Hybrid Implant. Audiol Neurotol. 2006;11: 63–68. doi: 10.1159/000095616 17063013

[3] Büchner A, Schüssler M, Battmer RD, Stöver T, Lesinski-Schiedat A, Lenarz T. Impact of low-frequency hearing. Audiology and Neurotology. Audiol Neurootol; 2009. pp. 8–13. doi: 10.1159/000206490 19390170

[4] Lenarz T, Stöver T, Buechner A, Lesinski-Schiedat A, Patrick J, Pesch J. Hearing conservation surgery using the hybrid-L electrode: Results from the first clinical trial at the Medical University of Hannover. Audiology and Neurotology. 2009. pp. 22–31. doi: 10.1159/000206492 19390172

[5] Brant JA, Ruckenstein MJ. Electrode selection for hearing preservation in cochlear implantation: A review of the evidence. World J Otorhinolaryngol—Head Neck Surg. 2016;2: 157–160. doi: 10.1016/j.wjorl.2016.08.002 29204561 PMC5698544

[6] Huarte RM, Roland JT. Toward hearing preservation in cochlear implant surgery. Curr Opin Otolaryngol Head Neck Surg. 2014;22: 349–352. doi: 10.1097/MOO.0000000000000089 25101938

[7] Miranda PC, Sampaio ALL, Lopes RAF, Ramos Venosa A, Oliveira CACP de. Hearing Preservation in Cochlear Implant Surgery. Int J Otolaryngol. 2014;2014: 1–6. doi: 10.1155/2014/468515 25276136 PMC4167950

[8] Jurawitz MC, Büchner A, Harpel T, Schüssler M, Majdani O, Lesinski-Schiedat A, et al. Hearing preservation outcomes with different cochlear implant electrodes: Nucleus® hybridTM-L24 and nucleus freedomTM CI422. Audiol Neurotol. 2014;19: 293–309. doi: 10.1159/000360601 25277083

[9] Sohmer H, Kinarti R, Gafni M. Source, au niveau de la membrane basilaire, du potentiel microphonique cochléaire enregistré par électrodes de surface chez l’homme. Electroencephalogr Clin Neurophysiol. 1980;49: 506–514. doi: 10.1016/0013-4694(80)90393-4 6158432

[10] Pappa AK, Hutson KA, Scott WC, Wilson JD, Fox KE, Masood MM, et al. Hair cell and neural contributions to the cochlear summating potential. Journal of Neurophysiology. 2019; 121:6 2163–2180. doi: 10.1152/jn.00006.2019 30943095 PMC6620691

[11] Zheng XY, Ding DL, McFadden SL, Henderson D. Evidence that inner hair cells are the major source of cochlear summating potentials. Hear Res. 1997;113: 76–88. doi: 10.1016/s0378-5955(97)00127-5 9387987

[12] Snyder RL, Schreiner CE. The auditory neurophonic: Basic properties. Hear Res. 1984;15: 261–280. doi: 10.1016/0378-5955(84)90033-9 6501114

[13] Ruben RJ, Sekula J, Bordley JE, Knickerbocker GG, Nager GT, Fisch U. XL Human cochlea responses to sound stimuli. Ann Otol Rhinol Laryngol. 1960;69: 459–479. doi: 10.1177/000348946006900214 14439841

[14] Wever EG, Bray CW. The nature of acoustic response. J Exp Psychol. 1930;13: 373–387. doi: 10.1037/h0075820

[15] Gibson WP. The Clinical Uses of Electrocochleography. Front Neurosci. 2017;11: 274. doi: 10.3389/fnins.2017.00274 28634435 PMC5437168

[16] Adunka O, Roush P, Grose J, Macpherson C, Buchman CA. Monitoring of Cochlear Function During Cochlear Implantation. Laryngoscope. 2006;116: 1017–1020. doi: 10.1097/01.mlg.0000217224.94804.bb 16735923

[17] Dalbert A, Pfiffner F, Röösli C, Thoele K, Sim JH, Gerig R, et al. Extra- and Intracochlear Electrocochleography in Cochlear Implant Recipients. Audiol Neurotol. 2015;20: 339–348. doi: 10.1159/000438742 26340649

[18] Mandalà M, Colletti L, Tonoli G, Colletti V. Electrocochleography during cochlear implantation for hearing preservation. Otolaryngol—Head Neck Surg (United States). 2012;146: 774–781. doi: 10.1177/0194599811435895 22291043

[19] Radeloff A, Shehata-Dieler W, Scherzed A, Rak K, Harnisch W, Hagen R, et al. Intraoperative Monitoring Using Cochlear Microphonics in Cochlear Implant Patients With Residual Hearing. Otol Neurotol. 2012;33: 348–354. doi: 10.1097/MAO.0b013e318248ea86 22377649

[20] Harris R, Cruise A, Gibson W, Bate K, Sanli H. Preliminary results and technique for electrophysiological intra-operative monitoring of residual hearing during cochlear implantation. Cochlear Implants Int. 2011;12: 209–215. doi: 10.1179/146701011X12950038111657 22251808

[21] Campbell L, Kaicer A, Sly D, Iseli C, Wei B, Briggs R, et al. Intraoperative Real-time Cochlear Response Telemetry Predicts Hearing Preservation in Cochlear Implantation. Otol Neurotol. 2016;37: 332–338. doi: 10.1097/MAO.0000000000000972 26859542

[22] Haumann S, Imsiecke M, Bauernfeind G, Büchner A, Helmstaedter V, Lenarz T, et al. Monitoring of the Inner Ear Function During and After Cochlear Implant Insertion Using Electrocochleography. Trends Hear. 2019;23: 233121651983356. doi: 10.1177/2331216519833567 30909815 PMC6435875

[23] Giardina CK, Brown KD, Adunka OF, Buchman CA, Hutson KA, Pillsbury HC, et al. Intracochlear electrocochleography: Response patterns during cochlear implantation and hearing preservation. Ear Hear. 2019;40: 833–848. doi: 10.1097/AUD.0000000000000659 30335669 PMC6534483

[24] Fitzpatrick DC, Campbell AT, Choudhury B, Dillon MP, Forgues M, Buchman CA, et al. Round window electrocochleography just before cochlear implantation: Relationship to word recognition outcomes in adults. Otol Neurotol. 2014;35: 64–71. doi: 10.1097/MAO.0000000000000219 24317211 PMC4447311

[25] Formeister EJ, McClellan JH, Merwin WH, Iseli CE, Calloway NH, Teagle HFB, et al. Intraoperative Round Window Electrocochleography and Speech Perception Outcomes in Pediatric Cochlear Implant Recipients. Ear Hear. 2015;36: 249–260. doi: 10.1097/AUD.0000000000000106 25259669

[26] Scott WC, Giardina CK, Pappa AK, Fontenot TE, Anderson ML, Dillon MT, et al. The compound action potential in subjects receiving a cochlear implant. Otol Neurotol. 2016;37: 1654–1661. doi: 10.1097/MAO.0000000000001224 27749750 PMC5242224

[27] Dalbert A, Huber A, Veraguth D, Roosli C, Pfiffner F. Assessment of Cochlear Trauma During Cochlear Implantation Using Electrocochleography and Cone Beam Computed Tomography. Otol Neurotol. 2016;37: 446–453. doi: 10.1097/MAO.0000000000000998 26945317

[28] Dalbert A, Pfiffner F, Hoesli M, Koka K, Veraguth D, Roosli C, et al. Assessment of Cochlear Function during Cochlear Implantation by Extra- and Intracochlear Electrocochleography. Front Neurosci. 2018;12: 18. doi: 10.3389/fnins.2018.00018 29434534 PMC5790789

[29] Koka K, Riggs WJ, Dwyer R, Holder JT, Noble JH, Dawant BM, et al. Intra-Cochlear Electrocochleography During Cochear Implant Electrode Insertion Is Predictive of Final Scalar Location. Otol Neurotol. 2018;39: e654–e659. doi: 10.1097/MAO.0000000000001906 30113557 PMC6097527

[30] O’Connell BP, Holder JT, Dwyer RT, Gifford RH, Noble JH, Bennett ML, et al. Intra- and Postoperative Electrocochleography May Be Predictive of Final Electrode Position and Postoperative Hearing Preservation. Front Neurosci. 2017;11: 291. doi: 10.3389/fnins.2017.00291 28611574 PMC5447029

[31] Adunka OF, Giardina CK, Formeister EJ, Choudhury B, Buchman CA, Fitzpatrick DC. Round window electrocochleography before and after cochlear implant electrode insertion. Laryngoscope. 2016;126: 1193–1200. doi: 10.1002/lary.25602 26360623 PMC5949050

[32] Koka K, Saoji AA, Litvak LM. Electrocochleography in Cochlear Implant Recipients With Residual Hearing. Ear Hear. 2017;38: e161–e167. doi: 10.1097/AUD.0000000000000385 27879487

[33] Kim JS, Tejani VD, Abbas PJ, Brown CJ. Postoperative Electrocochleography from Hybrid Cochlear Implant users: An Alternative Analysis Procedure. Hear Res. 2018;370: 304–315. doi: 10.1016/j.heares.2018.10.016 30393003 PMC6309996

[34] Verbist BM, Skinner MW, Cohen LT, Leake PA, James C, Boëx C, et al. Consensus panel on a cochlear coordinate system applicable in histologic, physiologic, and radiologic studies of the human cochlea. Otol Neurotol. 2010;31: 722–730. doi: 10.1097/MAO.0b013e3181d279e0 20147866 PMC2945386

[35] Dees G, van Hoof M, Stokroos R. A Proposed Method for Accurate 3D Analysis of Cochlear Implant Migration Using Fusion of Cone Beam CT. Front Surg. 2016;3. doi: 10.3389/fsurg.2016.00003 26835459 PMC4725264

[36] Dietz A, Gazibegovic D, Tervaniemi J, Vartiainen VM, Löppönen H. Insertion characteristics and placement of the Mid-Scala electrode array in human temporal bones using detailed cone beam computed tomography. Eur Arch Oto-Rhino-Laryngology. 2016;273: 4135–4143. doi: 10.1007/s00405-016-4099-x 27194346

[37] Stakhovskaya O, Sridhar D, Bonham BH, Leake PA. Frequency map for the human cochlear spiral ganglion: Implications for cochlear implants. JARO—J Assoc Res Otolaryngol. 2007;8: 220–233. doi: 10.1007/s10162-007-0076-9 17318276 PMC2394499

[38] Weder S, Bester C, Collins A, Shaul C, Briggs R, O’Leary S. Toward a Better Understanding of Electrocochleography: Analysis of Real-Time Recordings. Ear Hear. 2020; 41(6):1560–1567. doi: 10.1097/AUD.0000000000000871 33136631

[39] Kohllöffel LUE. Longitudinal amplitude and phase distribution of the cochlear microphonic (guinea pig) and spatial filtering. J Sound Vib. 1970;11: 325–334. doi: 10.1016/S0022-460X(70)80036-0

[40] Bester C, Weder S, Collins A, Dragovic A, Brody K, Hampson A, et al. Cochlear microphonic latency predicts outer hair cell function in animal models and clinical populations. Hear Res. 2020 Dec;398:108094. doi: 10.1016/j.heares.2020.108094 33099252

[41] Avci E, Nauwelaers T, Lenarz T, Hamacher V, Kral A. Variations in microanatomy of the human cochlea. J. Comp. Neurol. 2014, 522: 3245–261. 10.1002/cne.23594 24668424 PMC4265794

